# Gene expression analysis in formalin fixed paraffin embedded melanomas is associated with density of corresponding immune cells in those tissues

**DOI:** 10.1038/s41598-020-74996-9

**Published:** 2020-10-27

**Authors:** Minyoung Kwak, Gulsun Erdag, Craig L. Slingluff

**Affiliations:** 1 Department of Surgery, University of Virginia, P.O. Box 800709, Charlottesville, VA 22908-0709 USA; 2 Department of Surgery, SUNY Downstate Medical Center, Brooklyn, NY USA

**Keywords:** Melanoma, Cancer microenvironment

## Abstract

Immune cell infiltrates in melanoma have important prognostic value. Gene expression analysis may simultaneously quantify numbers and function of multiple immune cell subtypes in formalin-fixed paraffin-embedded (FFPE) tissues. Prior studies report single gene expression can represent individual immune cell subtypes, but this has not been shown in FFPE melanomas. We hypothesized that gene expression profiling of human melanomas using a new RNA expression technology in FFPE tissue would correlate with the same immune cells identified by immunohistochemistry (IHC). This retrospective study included melanoma specimens analyzed by IHC on tumor tissue microarray (TMA) cores and by gene expression profiling with EdgeSeq Immuno-Oncology Assay using qNPA technology on the corresponding tumors. Standardized gene expression levels were analyzed relative to enumerated cells by IHC using Spearman rank test to calculate r-values. Multivariate analysis was performed by Kruskal–Wallis test. 119 melanoma specimens had both IHC and gene expression information available. There were significant associations between the level of gene expression and its quantified IHC cell marker for CD45^+^, CD3^+^, CD8^+^, CD4^+^, and CD20^+^ cells (all *p* < 0.001). There were also significant associations with exhaustion markers FoxP3^+^, PD-1^+^, and PD-L1^+^ (all *p* ≤ 0.0001). This new qNPA technology is useful to quantify intratumoral immune cells on FFPE specimens through RNA gene expression in metastatic melanoma. As previous studies have shown on other solid human tumors, we also confirm that the expression level of a single gene may be used to represent a single IHC immune cell marker in melanoma.

## Introduction

Gene expression profiling of cancer tissue offers a window into the immunobiology of the tumor microenvironment (TME) of human cancers. Several gene expression profiles have been reported to predict clinical responses to systemic immunotherapy^1–5^ and to be prognostic for patients with a range of cancers, including melanoma^6–9^. A more traditional approach for defining immunologic features of the TME has been to quantify numbers of selected intratumoral immune cells within the TME which has also been found to predict responses to certain immunotherapies or overall prognosis from melanoma^6,7,10–12^ and other cancers^13,14^. Pathologists can report immune cell density on formalin-fixed paraffin-embedded (FFPE) tissue using hematoxylin and eosin (H&E) stains or immunohistochemistry (IHC), but these modalities are cumbersome and costly to integrate into routine clinical practice as quantitative assays. Furthermore, those traditional approaches limit the simultaneous analysis of multiple immune markers and can be tedious and time consuming, with automated techniques still years away from becoming fully integrated into routine clinical practice^15,16^. In research environments, flow cytometry can accurately define immune cell subsets but this requires viable single cell suspensions which are not routinely available and cannot be performed on FFPE specimens which are most typically available in clinical settings. Gene expression profiling measures mRNA levels in tissue, and traditional methods are most accurate when the tissue is preserved in RNAlater or flash frozen within about 15 min. This allows tissue samples collected in selected research studies available for gene analysis but not with FFPE tissues handled routinely in pathology laboratories. On the other hand, newer technologies have overcome some of these challenges to enable gene expression analysis on FFPE tissues; however, these remain fairly new techniques^17–20^.

A few studies have validated these techniques by identifying genes specific to different immune cells and showing associations between expression levels of these genes^21^ and respective cell populations in purified in vitro assays^22,23^. One study assessed correlations between these same gene markers from tumor lysates with flow cytometry of peripheral blood mononuclear cell (PBMC) and also performed a limited analysis of associations between CD3^+^ and CD8^+^ cells by IHC in 19 ovarian FFPE cancer specimens^24^. To our knowledge, however, no previous study has assessed associations of gene expression in FFPE tissue with a wider range of lymphoid and myeloid markers and checkpoint blockade molecules in human solid tumors. We have addressed this in the present study for over 100 FFPE human melanomas.

Gene sequencing techniques on FFPE tissue are generally performed through real-time quantitative PCR (RT-qPCR), yet RNA extracted from FFPE tissue can be degraded, can be crosslinked to other proteins, or can lack the poly(A) tail needed to generate cDNA, all potentially leading to inaccurate outputs and poor yields of transcript^25–27^. The unique advantage proposed by the quantitative nuclease protection assay (qNPA) with Next Generation Sequence (NGS) technology is that it does not require nucleic acid extraction, cDNA synthesis, or gene amplification, thereby reducing the risk of RNA degradation and biases due to molecular artifacts, while also requiring less tissue^28–31^. The qNPA technology creates DNA to RNA hybridization, called the nuclease protection probe (NPP), which is then detected and sequenced using standard next-generation sequencing protocols after S1 nuclease is added to digest excess non-hybridized DNA probes and non-hybridized RNA^28,29,31^. This technology has previously been validated on human lymphoma and canine cancers^28,30^, but not on human solid cancers such as melanoma. In this present study, we assessed the validity of qNPA technology on human melanomas by comparing the levels of gene expression for single immune cell marker subtypes^21,22,24^ with these same immune cells identified by IHC performed on the same FFPE tumors. If valid, these gene expression profiles would reflect the densities of intratumoral immune cells measured by IHC and allow the simultaneous measurement of multiple immune cell markers within the TME even when limited amounts of FFPE tumor tissue are available.

## Methods

### Patients, tumor microarrays and immunohistochemistry

Adult patients diagnosed with melanoma had previously been identified by the Anatomic Pathology Software System in the Department of Pathology, University of Virginia and archival tumor specimens from 1982 to 2007 were sampled to create a melanoma tissue microarray. All methods were performed in accordance with the appropriate guidelines and regulations from the University of Virginia IRB (IRB #10598, 10803, 13310) with waiver of consent due to retrospective design of study. Melanoma specimens with ample tissue and appropriate clinical follow-up were selected for creation of a melanoma tissue microarray (TMA), as previously described^10^. Briefly, areas of FFPE tumor tissue blocks from these surgical specimens were identified by a pathologist on H&E slides to identify central areas within tumor cell nests for TMA sampling. TMAs were constructed from 3 to 4 cores (1.0 mm diameter) through the tumor regions of each specimen from the selected FFPE blocks. No duplicate samples were represented from the same surgical specimen. Control tissues from liver, spleen, placenta, and kidney were included in each TMA block. TMA tissue sections were previously evaluated for immune cell infiltrates by immunohistochemistry (IHC) staining using standard protocols. Positive controls included lymph nodes (LN) and placenta for CD34 antibody. Negative control slides used PBS instead of primary antibody, with other conditions constant. Immune cells within each core were enumerated by a surgical pathologist^10^.

### Immunotype and immune cell density assessments

We previously reported that melanoma can be categorized into 3 Immunotype groups based on the extent and patterns of CD45^+^ immune cell infiltrates relative to CD34^+^ vasculature^10^. In each core, intratumoral immune cells were scored as 1 when immune cells (CD45^+^) were absent or sparse (no more than 50 immune cells per 1 mm diameter core); 2 when intratumoral immune cells were present but were limited to perivascular cuffing around intratumoral blood vessels; 3 when immune cells were diffusely present among tumor cells in different areas of the core. Mean scores were calculated for each tumor sample and three Immunotype groups were created to reflect these three patterns of infiltration: Immunotype A for mean scores < 1.75, Immunotype B for 1.75–2.4, and Immunotype C for ≥ 2.5. Additional details have been reported^10^. The TMA samples were also evaluated for the density of a range of immune cells including CD8 (CD8^+^ T cells), CD4 (CD4^+^ T cells), CD56 (NK cells), CD20 (B cells), CD138 (plasma cells), DC-LAMP (mature dendritic cells), CD163 (M2 macrophages), FoxP3 (regulatory T cells), as well as for cells expressing PD-1, PD-L1, and PD-L2^32^.

For each tumor specimen, mean values of the quantified immune cell populations were recorded among the triplicate or quadruplicate cores for each tumor sample, as previously described^10^. These cores from each tumor sample were also evaluated for homogeneity. When the Immunotype scores were consistent across all three or four replicates, these samples have been considered homogeneous for immunotype. However, when they were not all consistent, these samples have been considered heterogeneous for immunotype.

### Quantitative nuclease protection assay

Among the FFPE tissue blocks that had been analyzed by IHC in the TMAs, 119 were also evaluated in the present study for expression of immune-related genes (IRB #17816). These included 112 metastases (94%) and 7 primary melanomas (6%). One five micrometer section of each FFPE tumor specimen was submitted to HTG Molecular (HTG Molecular, Tucson, AZ). The HTG EdgeSeq Immuno-Oncology Assay was performed for gene expression analysis, using 558 probes with 15 housekeeper genes, 5 negative and 4 positive processor controls. For this assay, functional DNA Nuclease Protection Probes (NPPs) are flanked by universal wing sequences that are hybridized to the target RNAs, which can be both soluble and cross-linked in the biological matrix. Universal DNA wingmen probes are hybridized to the wings to prevent S1 nuclease digestion. S1 nuclease is added to digest excess non-hybridized DNA probes and non-hybridized RNA. This reaction then results in a stoichiometric quantity of fully intact NPPs:RNA hetero-duplexes of interest. The released and labeled DNA protection proves are concentrated, pooled, and sequenced using standard next-generation sequencing (NGS) protocols on the Illumina NextSeq™ platform. Gene expression data from the NGS instrument are processed and reported through the HTG EdgeSeq host system software. Data was standardized through a procedure that log-transformed Counts Per Million (CPM) and adjusted for total reads within a sample^33^.

To evaluate concordance of cell enumeration by IHC with those identified by gene expression, genes chosen for study were those previously shown, through meta-analyses, to be expressed selectively on certain immune cell populations^21,22,24^ and for which gene primers were available in the HTG EdgeSeq Immuno-Oncology Assay. Several markers of immune cell exhaustion were also selected for study^34^.

### Statistics

Statistical comparisons among multiple subgroups were performed using the Kruskall-Wallis test. Correlation coefficients (r values) were performed using the Spearman rank test. Statistical analysis was performed using Prism 8.0 (Graphpad Software Inc., San Diego, CA). *p* values < 0.05 were considered significant.

### Ethics approval and consent to participate

This research study was conducted retrospectively from data obtained for clinical purposes. IRB approved study (University of Virginia IRB numbers 10598, 10803, 13310, and 17816) with waiver of consent due to retrospective design of study.

## Results

### Establishing baseline differences of intratumoral immune cell populations using the Immunotype Score by IHC

**Figure 1:**
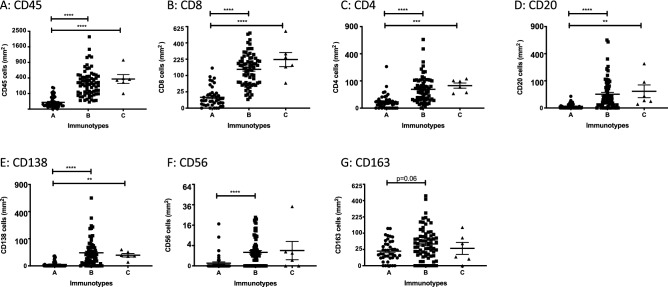
Intratumoral populations of immune cell that make up Immunotype A, B, and C melanoma tumors (n = 119). Immunotype Groups were established based on location and mean number of immune cells within tumor nests. Intratumoral immune cells in Immunotype A (n = 42) were very low, Immunotype B (n = 71) were limited to perivascular cuffing, and Immunotype C (n = 6) were highly infiltrated. Each panel shows quantified values by IHC of each immune cell subtype among the three Immunotype Groups. X axis shows the three different Immunotype Groups. Y axis shows the number of cells per mm^2^ on a square root scale. Bars represent the means and standard errors of the mean for each group. Statistical analysis based on the Kruskall–Wallis test. *p* value < 0.05 (*), < 0.01 (**), < 0.001 (***), < 0.0001 (****).

We obtained gene expression analysis data on FFPE tumor blocks for which immune cell densities were previously measured by IHC on melanoma TMAs^10^. Of the 183 tumor specimens previously analyzed by IHC, we included FFPE blocks that were still available and included sufficient tumor for gene expression analysis. A total of 119 tumor specimens had both IHC and gene expression data from the same FFPE block, and included both primary (n = 7, 6%) and metastatic (n = 112, 94.1%) tumors. No duplicate primary or metastatic tumor specimens were represented. To categorize the patterns of intratumoral immune cell populations among the 119 tumors, we used the Immunotype Score from a previous analysis that was based on immune cell density and distribution relative to the tumor and proximity to intratumoral vasculature^10^. The Immunotype Scores represent a continuous variable from 1.0 to 3.0. These scores were previously categorized into Immunotype Groups, where Immunotype A tumors (n = 42, 35%) have very little to no immune infiltrates, Immunotype B tumors (n = 71, 60%) have greater immune infiltrate but mostly limited to perivascular cuffing, and Immunotype C tumors (n = 6, 5%) were heavily infiltrated. The intratumoral cell densities of immune cell subtypes including CD45^+^ immune cells, CD8^+^ T cells, CD4^+^ T cells, CD20^+^ B cells, CD138^+^ plasma cells, CD56^+^ natural killer (NK) cells, and CD163^+^ macrophages/monocytes among the three different Immunotype Groups are shown in Fig. 1. The vast majority of Immunotype A tumors have less than fifty CD45^+^ immune cells per mm^2^, while Immunotype B and C have significantly greater numbers (Fig. 1A). Similar differences are also evident with CD8^+^ and CD4^+^ T cells, CD20^+^ and CD138^+^ cells (all *p* < 0.01, Fig. 1B–E). CD56^+^ cells were greater in Immunotype B than A (*p* < 0.0001), but the difference between Immunotype C versus A did not reach statistical significance (Fig. 1F). There was no difference in CD163^+^ cells among the groups, though CD163^+^ cells in Immunotype B did trend towards significance (*p* = 0.06, Fig. 1G) compared to Immunotype A.

### Gene expression by qNPA on FFPE melanoma specimens was significantly associated with intratumoral immune cell density by IHC

**Figure 2:**
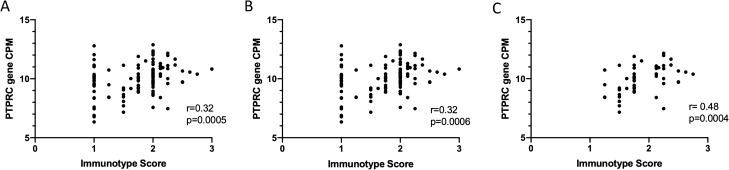
Scatter plots of expression level for immune cell gene marker and the Immunotype Scores as determined by IHC. Immunotype Scores categorized Immunotype Groups based on mean values of intratumoral immune cells measured on tissue microarray by IHC that included triplicate or quadruplicate tumor cores: Immunotype A for mean scores < 1.75, Immunotype B for 1.75–2.4, and Immunotype C for ≥ 2.5. (**A**) Includes all tumors (n = 119). (**B**) Includes metastatic tumors only (n = 112). (**C**) Includes heterogenous tumors with inconsistent tumor cores observed by IHC (n = 50). X axis is Immunotype Score. Y axis is level of PTPRC gene expression represented by standardized counts per million (CPM). Correlation coefficients shown as r values.

We then analyzed the qNPA gene expression data from the same FFPE specimens to compare gene expression with the population of immune cell subsets determined by the Immunotype Score. To represent the gene for CD45^+^ immune cells, we used the PTPRC gene as described in other studies^21,24^. For all tumors, there was a highly significant association between the Immunotype Score and the expression of PTPRC (r = 0.32, *p* = 0.0005, Fig. 2A, Table 1). Tumor subgroup analysis also showed the correlation coefficients between gene expression of the 112 metastatic tumors alone and the Immunotype Score were similar to the overall group (r = 0.32, *p* = 0.0006, Fig. 2B). The number of primary tumors was too small to evaluate meaningfully, but among those 7 primary tumors, the r value for association of Immunotype Score with PTPRC did not reach statistical significance and did not change with different densities of immune cells in Immunotype A (n = 4) primary melanomas or in Immunotype B + C (n = 3) primary tumors (data not shown).

**Table 1:** Correlation coefficients (r values) for the level of gene expression of immune cell markers and enumerated immune cell subtypes identified by IHC.

Immune cell type	Protein assessed by IHC	Gene	All tumors n = 119		Immunotype A tumors n = 42		Immunotype B + C tumors n = 77	
			r	*p*	r	*p*	r	*p*
Hematopoietic cells	CD45	PTPRC	0.37	< 0.0001	− 0.14	0.39	0.37	0.0009
T cells	CD3	CD3D	0.54	< 0.0001	− 0.05	0.77	0.64	< 0.0001
CD8 T cells	CD8	CD8A	0.67	< 0.0001	− 0.03	0.87	0.71	< 0.0001
CD4 T cells	CD4	CD4	0.32	0.0004	0.23	0.15	0.16	0.17
B Cells	CD20	CD19	0.33	0.0003	− 0.03	0.87	0.43	< 0.0001
B cells	CD20	MS4A1	− 0.07	0.45	−	−	−	−
NK cells	CD56	FCGR3A	0.22	0.02	0.06	0.72	0.04	0.76
NK cells	CD56	DUSP4	0.05	0.63	−	−	−	−
T regulatory cells	FoxP3	FOXP3	0.35	< 0.0001	0.28	0.08	0.35	0.0016
Exhaustion	PD-1	PDCD1	0.34	0.0001	0.03	0.84	0.41	0.0002
Exhaustion	LAG-3	LAG3	0.46	< 0.0001	0.20	0.20	0.32	0.005
Exhaustion	PD-L1	CD274	0.5	< 0.0001	0.42	0.005	0.49	< 0.0001
Exhaustion	PD-L2	PDCD1LG2	0.17	0.063	–	–	–	–

Analysis was stratified by all tumors (n = 119), only tumors with Immunotype A (n = 42) and tumors with Immunotype B or C (n = 77). R values calculated by Spearman Rank tests between standardized gene expression levels and cell counts per mm^2^ by IHC. (–) signifies analysis could not be performed.

The Immunotype scores established by IHC were consistent among all cores for the majority of the included tumors, but 50 (42%) specimens were heterogeneous in the Immunotype patterns for immune infiltrate among the 3–4 TMA cores evaluated for the same tumor specimen. Despite this heterogeneity found on IHC analysis, the mean density of intratumoral CD45^+^ cells was strongly and significantly associated with the expression of PTPRC even in this subset (r = 0.48, *p* = 0.0004, Fig. 2C).

### Gene expression by qNPA was significantly associated with densities of immune cell subtypes identified by IHC

**Figure 3:**
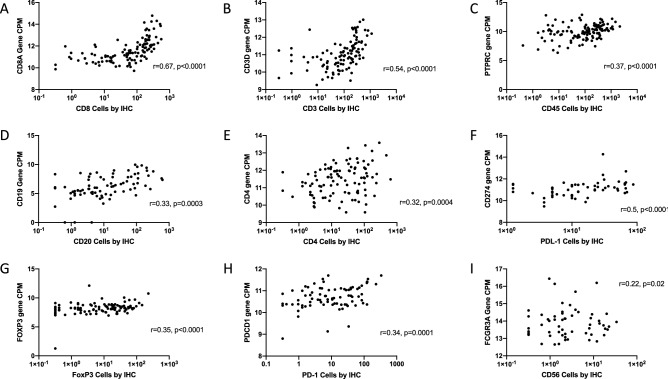
Scatter plots comparing immune cell subtypes by immune cell gene marker expression level versus its associated cell marker quantified by IHC of all tumors (n = 119). X axis represents cell densities (per mm^2^) in tumor by IHC. Y axis represents the level of single RNA gene expression for the respective immune cell subtype, reported as standardized counts per million (CPM). Correlation coefficients shown as r values and calculated by Spearman rank tests.

Tumor microenvironments vary in their composition of T cell subsets, B cells, myeloid cells, and other subsets, and these may have prognostic relevance or value in predicting response to immune therapies. To investigate whether qNPA gene expression profiling of FFPE tumors may provide comparable data to those obtained with enumeration of the cell subsets by IHC, we selected single genes to identify individual immune cell subtypes as reported previously^21,22,24^, and assessed whether expression of these genes was significantly associated with counts of those cells per mm^2^. Expression levels of these genes were most strongly and significantly correlated with the density of CD8^+^ T cells (CD8A gene, r = 0.67, *p* < 00001, Fig. 3A). Highly significant associations between gene expression and enumerated intratumoral cell subtypes (all *p* < 0.001, Fig. 3B–E) were also observed for CD3^+^ cells (CD3D gene, r = 0.54), CD45^+^ cells (PTPRC gene, r = 0.37), CD20^+^ B cells (CD19 gene, r = 0.33), CD4^+^ T cells (CD4 gene, r = 0.32). Similar results were also found with exhaustion gene markers and their concordant IHC markers (all *p* ≤ 0.0001, Fig. 3F–H) including PD-L1^+^ (CD274, r = 0.5), FoxP3^+^ cells (FOXP3 gene, r = 0.35) and PD-1^+^ cells (PDCD1 gene, r = 0.34).

Three gene markers for NK cells were included in the current RNA assay, among 31 gene candidates previously used in the literature^21,22,24^. Among these three NK cell gene candidates (DUSP4, BCL2, SPN), we did not find significant correlations with CD56^+^ NK cell density by IHC (data not shown). Thus, we chose to evaluate another NK-associated gene, FCG3A^35^, and identified a relatively weak, yet still significant, association with CD56^+^ NK cell density (r = 0.22, *p* = 0.02; Fig. 3I). We had assessed macrophages by IHC using the CD163^+^ marker, which is expressed only by macrophages and monocytes and is increased in chronic inflammation and commonly associated with M2 macrophages^36,37^. Of 50 macrophage-associated genes reported^21,22,24^, the qNPA assay included 5: CD68, ATG7, CLEC5A, CYBB, FN1, MSR1. Associations of each of these with CD163^+^ density by IHC were all insignificant (data not shown). We did not assess chemokines as possible gene cell markers candidates previously used in the literature due to the possible confounding effect of the pro-inflammatory TME (Kwak, submitted under review).

Next, we hypothesized associations between gene expression and IHC measures for immune cell infiltrates would be stronger for those tumors that had greater populations of intratumoral immune cells. Subgroup analyses were performed of Immunotypes B + C tumors (n = 77), and of Immunotype A (n = 42) tumors. Those analyses did show that, among Immunotype B + C tumors, the correlation coefficients were increased between IHC counts for CD3^+^, CD8^+^, CD20^+^, and PD-1^+^ and their respective gene expression levels, compared to the data from the full dataset (r = 0.41 to 0.71, all *p* < 0.0005, Table 1). Also consistent with our hypothesis, these same associations lost significance within the subgroup of the Immunotype A tumors alone. Within the immunotype B + C subgroup, associations of cell density and gene marker expression were similar to those in the full dataset for FoxP3^+^, PD-1^+^ and PD-L1^+^ cells, but lost significance for CD56^+^ and for CD4^+^ cells.

## Discussion

Gene expression analysis provides a valuable tool to assess immune cell populations in tumor microenvironments. Most human tissues available for analysis are FFPE; thus, new technologies for gene expression analysis in these tissues have opened the door to evaluate archived specimens and those collected as part of clinical care. However, several factors can modify those measures, and thus can impact their accuracy. Formalin fixation leads to cross-linking and degradation of mRNA and can have differential effects on different mRNAs^38^. Tissue autolysis at room temperature, prior to fixation, can also affect mRNA levels, especially after 24 h^38^. Newer technologies overcome some of these limitations but warrant additional validation in human tissues. Also, a potential limitation of gene expression analysis for quantitation of immune cells in tumor tissue is that the expression levels of some marker genes (e.g., CD8^+^ T cells^39^) are not constant per cell, and thus may be modulated by immune activation or other features of the microenvironment so that their expression level may be an imperfect correlate of cell number. Finally, spatial variations in immune cell distribution can lead to differences in results based on sampling different areas of the tumor. In a prior study, quantitation of T cells, and of CD8^+^ T cells in particular by flow cytometry of PBMC samples, was compared to quantitation based on gene expression in FFPE tissue with Nanostring technology, with findings that the correlation r values were 0.657 (*p* = 0.002) and 0.481 (*p* = 0.037), respectively^24^. The present study has provided an opportunity to assess similar correlations with a different technology for a wider range of immune cells in FFPE solid tumor tissue.

Assessing the qNPA technology, we found that expression of the PTPRC gene was significantly associated with the immune infiltration pattern (Immunotype score), though correlation coefficients were only 0.32 overall, and somewhat higher (r = 0.48) for the subset of tumors heterogeneous for the Immunotype by IHC. Tumor heterogeneity is a common features of cancers; so, sampling of selected tumor areas may not reflect the entire tumor. We have found that immune cell infiltrates in melanoma metastases strongly predict clinical outcome, when assessed with mean values derived from 3 to 4 core samples of central tumor areas^10^. In prior studies, we have evaluated how heterogeneity impacts the relevance of CD8^+^ T cell counts in small tumor samples. We have shown that a single core sample often misrepresents the CD8^+^ T cell density of the whole tumor, and that there can be significant differences in CD8^+^ T cell densities among pairs of metachronous tumors. However, a large biopsy of one tumor may be representative of multiple synchronous metastases^40^. Also by modeling different ways to sample a lung cancer, we found that different sampling strategies yield different densities of CD8^+^ infiltrate, but that the values most concordant with whole tumor counts were predicted by a random core sampling of the tumor or sampling of the tumor center^41^. These studies support our approach of using triplicate or quadruplicate 1 mm diameter cores from each tumor specimen as a reasonable representation of the tumor. Each tumor specimen was from a specific surgical resection. Multiple metastatic sites were not analyzed in this study, but 42% of tumors in our analysis had heterogenous tumor cores on IHC, yet data from these tumors were still very significantly associated with gene expression of immune cells as a measure of melanoma Immunotype.

The densities of various immune cell sub-populations by IHC, were also significantly associated with expression of the cell-associated gene, with correlation coefficients of 0.67 and 0.54 for CD8^+^ T cells and CD3^+^ T cells, respectively. These are comparable to, or slightly better than, similar correlation coefficients for the Nanostring approach^24^. In addition, significant positive correlations were observed for PTPRC expression with CD45^+^ cells by IHC. This was also true for B cells and CD4^+^ T cells, as well. Significant correlation was also observed for CD56^+^ cells and FCGR3A expression but with lower correlation coefficient and wide variance across the population. There was a significant association for FoxP3^+^ cells, but the gene expression values were limited to a narrow range, making the findings less useful than the others for extrapolation to actual cell counts. Interestingly, expression of PD1^+^ and of PDL1^+^ were closely correlated with the counts of cells expressing them, by IHC. Thus, these data support the value of gene expression analysis by the qNPA method for evaluation of sets of tumors for all of these markers, but patient-level data appears to be more reliable for only a subset of these immune cell markers.

This study was limited in part by the fact that the IHC studies were performed on 3–4 cores (1 mm diameter) collected from tumor cell nests, rather than from peritumoral regions, whereas whole tissue sections were sent for the gene expression analysis. Thus, the associations of cell counts by IHC and corresponding gene expression may reflect some differences in the portion of the tumor that was sampled for each. Most associations were stronger among tumors with greater than 50 immune cells per mm^2^ (Immunotype B + C tumors) than among those without significant infiltrates within tumor nodules (Immunotype A tumors), which is likely explained in part by disproportionately high impact of peritumoral immune cells when tumor cell nests exclude immune cells, as is observed with Immunotype A tumors. Immune cell heterogeneity within tumors may contribute to decreased associations between gene expression analysis and IHC quantification^40^. In particular, collections of immune cells present not within the tumor itself but within tertiary lymphoid structures (TLS) on the peripheral margins of tumor^42,43^ may have been represented by the gene expression analyses, but not by IHC of tumor cores taken from the center of tumor nodules. Other possible factors that could impact association between gene expression and IHC analysis are differences in time course for preservation of each tumor specimen and the length of time each specimen was stored, especially if protein is more stable than mRNA. Considering these limitations, the strongly significant associations for multiple immune cell markers is more remarkable and supports use and further investigation with this qNPA technology.

Of all the immune cell subtypes investigated, we did not find a gene marker in this particular qNPA Immuno-Oncology Assay panel that was significantly associated with density of CD163^+^ cells by IHC. This may reflect low numbers of CD163^+^ cells in the tumors evaluated, or the need for other gene markers for CD163^+^ cells. Previous studies have shown correlations with 50 single gene markers as possible candidates to capture macrophage cell populations in solid tumors, and the current assay included only 5 of them^21,22,24^. We did not customize this assay for our analysis and so other gene markers such as CD163, MS4A4A, ARG1, and CD84 genes^21,24^ could also be assessed in future studies.

Overall, the use of qNPA technology on human metastatic solid tumors such as melanoma is supported by the present analysis to identify FFPE tumor specimens as having high- or low- densities of varied immune cell infiltrates. As previous studies have shown on solid human tumors, we also confirm that in selected cases, the expression level of a single gene may be used to represent a single IHC immune cell marker in melanoma. Even when heterogenous tumor samples were found through analysis of 3–4 individual TMA cores, the intratumoral immune cell populations were still highly significantly associated with the level of single gene expression. Thus, quantification of multiple immune biomarkers simultaneously using qNPA technology may have significant value in future analysis of the TME, and possibly on peritumoral TLS, even with the use of small FFPE tumor specimens.

## References

[1] Jiang P (2018). Signatures of T cell dysfunction and exclusion predict cancer immunotherapy response. Nat. Med..

[2] Chen P-L (2016). Analysis of immune signatures in longitudinal tumor samples yields insight into biomarkers of response and mechanisms of resistance to immune checkpoint blockade. Cancer Discov..

[3] Herbst RS (2014). Predictive correlates of response to the anti-PD-L1 antibody MPDL3280A in cancer patients. Nature.

[4] Ji R-R (2012). An immune-active tumor microenvironment favors clinical response to ipilimumab. Cancer Immunol. Immunother..

[5] Snyder A (2014). Genetic basis for clinical response to CTLA-4 blockade in melanoma. N. Engl. J. Med..

[6] Clemente CG (1996). Prognostic value of tumor infiltrating lymphocytes in the vertical growth phase of primary cutaneous melanoma. Cancer.

[7] Tumeh PC (2014). PD-1 blockade induces responses by inhibiting adaptive immune resistance. Nature.

[8] Galon J, Fridman WH, Pages F (2007). The adaptive immunologic microenvironment in colorectal cancer: a novel perspective. Cancer Res..

[9] Zhang L (2003). Intratumoral T cells, recurrence, and survival in epithelial ovarian cancer. N. Engl. J. Med..

[10] Erdag G (2012). Immunotype and immunohistologic characteristics of tumor-infiltrating immune cells are associated with clinical outcome in metastatic melanoma. Cancer Res..

[11] Fridman WH, Pagès F, Sautès-Fridman C, Galon J (2012). The immune contexture in human tumours: impact on clinical outcome. Nat. Rev. Cancer.

[12] Ascierto PA (2017). Future perspectives in melanoma research “Melanoma Bridge”, Napoli, November 30^th^–3rd December 2016. J. Transl. Med..

[13] Galon J (2016). Immunoscore and Immunoprofiling in cancer: an update from the melanoma and immunotherapy bridge 2015. J. Transl. Med..

[14] Galon J (2006). Type, density, and location of immune cells within human colorectal tumors predict clinical outcome. Science.

[15] Shirazi SH, Naz S, Razzak MI, Umar AI, Zaib A, Dey N (2018). Chapter 2—automated pathology image analysis. Soft Computing Based Medical Image Analysis.

[16] NP Group (2017). Histopathology is ripe for automation. Nat. Biomed. Eng..

[17] Trejo CL (2019). Extraction-free whole transcriptome gene expression analysis of FFPE sections and histology-directed subareas of tissue. PLoS ONE.

[18] Yeri A (2018). Evaluation of commercially available small RNASeq library preparation kits using low input RNA. BMC Genom..

[19] Tsang H-F (2017). NanoString, a novel digital color-coded barcode technology: current and future applications in molecular diagnostics. Expert Rev. Mol. Diagn..

[20] Li J, Fu C, Speed TP, Wang W, Symmans WF (2018). Accurate RNA sequencing from formalin-fixed cancer tissue to represent high-quality transcriptome from frozen tissue. JCO Precis. Oncol..

[21] Bindea G (2013). Spatiotemporal dynamics of intratumoral immune cells reveal the immune landscape in human cancer. Immunity.

[22] Newman AM (2015). Robust enumeration of cell subsets from tissue expression profiles. Nat. Methods.

[23] Şenbabaoğlu, Y. *et al.* The landscape of T cell infiltration in human cancer and its association with antigen presenting gene expression. *bioRxiv*. 10.1101/025908 (2015).

[24] Danaher P (2017). Gene expression markers of tumor infiltrating leukocytes. J. Immuno Ther. Cancer.

[25] Srinivasan M, Sedmak D, Jewell S (2002). Effect of fixatives and tissue processing on the content and integrity of nucleic acids. Am. J. Pathol..

[26] Abrahamsen HN, Steiniche T, Nexo E, Hamilton-Dutoit SJ, Sorensen BS (2003). Towards quantitative mRNA analysis in paraffin-embedded tissues using real-time reverse transcriptase-polymerase chain reaction: a methodological study on lymph nodes from melanoma patients. J. Mol. Diagn..

[27] Cronin M (2004). Measurement of gene expression in archival paraffin-embedded tissues: development and performance of a 92-gene reverse transcriptase-polymerase chain reaction assay. Am. J. Pathol..

[28] Davis B, Schwartz M, Duchemin D, Carl Barrett J, Post G (2017). Validation of a multiplexed gene signature assay for diagnosis of canine cancers from formalin-fixed paraffin-embedded tissues. J. Vet. Intern. Med..

[29] Bourzac KM (2011). A high-density quantitative nuclease protection microarray platform for high throughput analysis of gene expression. J. Biotechnol..

[30] Roberts RA (2007). Quantitative nuclease protection assay in paraffin-embedded tissue replicates prognostic microarray gene expression in diffuse large-B-cell lymphoma. Lab. Invest..

[31] Martel RR (2002). Multiplexed screening assay for mRNA combining nuclease protection with luminescent array detection. Assay Drug Dev. Technol..

[32] Obeid JM (2016). PD-L1, PD-L2 and PD-1 expression in metastatic melanoma: correlation with tumor-infiltrating immune cells and clinical outcome. Oncoimmunology.

[33] Law CW, Chen Y, Shi W, Smyth GK (2014). voom: precision weights unlock linear model analysis tools for RNA-seq read counts. Genome Biol..

[34] Wherry EJ (2011). T cell exhaustion. Nat. Immunol..

[35] Mahaweni NM (2018). A comprehensive overview of FCGR3A gene variability by full-length gene sequencing including the identification of V158F polymorphism. Sci. Rep..

[36] Edin S (2012). The distribution of macrophages with a M1 or M2 phenotype in relation to prognosis and the molecular characteristics of colorectal cancer. PLoS ONE.

[37] Buechler C (2000). Regulation of scavenger receptor CD163 expression in human monocytes and macrophages by pro- and antiinflammatory stimuli. J. Leukoc. Biol..

[38] von Smolinski D, Leverkoehne I, von Samson-Himmelstjerna G, Gruber AD (2005). Impact of formalin-fixation and paraffin-embedding on the ratio between mRNA copy numbers of differently expressed genes. Histochem. Cell Biol..

[39] Xiao Z, Mescher MF, Jameson SC (2007). Detuning CD8 T cells: down-regulation of CD8 expression, tetramer binding, and response during CTL activation. J. Exp. Med..

[40] Obeid JM, Hu Y, Erdag G, Leick KM, Slingluff CL (2017). The heterogeneity of tumor-infiltrating CD8+ T cells in metastatic melanoma distorts their quantification: how to manage heterogeneity?. Melanoma Res..

[41] Obeid JM, Wages NA, Hu Y, Deacon DH, Slingluff CL (2017). Heterogeneity of CD8(+) tumor-infiltrating lymphocytes in non-small-cell lung cancer: impact on patient prognostic assessments and comparison of quantification by different sampling strategies. Cancer Immunol. Immunother..

[42] Zhu G (2017). Tumor-associated tertiary lymphoid structures: gene-expression profiling and their bioengineering. Front. Immunol..

[43] Engelhard VH (2018). Immune cell infiltration and tertiary lymphoid structures as determinants of antitumor immunity. J. Immunol..

